# Dysbiosis of the Gut–Lung Axis and Its Immune Correlates During Pulmonary *Cryptococcus neoformans* Infection

**DOI:** 10.3390/jof12030163

**Published:** 2026-02-25

**Authors:** Jing Fan, Shujun Liu, Huijiao Zhang, Changzhong Jin, Nanping Wu

**Affiliations:** Jinan Microecological Biomedicine Shandong Laboratory, Building 1, Jinan Medical and Health Science and Technology Innovation Industrial Park, No. 288, Jiqi Road, Huayin District, Jinan 250021, China; fanjing@jnl.ac.cn (J.F.); liushujun@jnl.ac.cn (S.L.); zhanghuijiao@jnl.ac.cn (H.Z.)

**Keywords:** fungal infection, microbiome, gut–lung axis

## Abstract

*Cryptococcus neoformans* is a major fungal pathogen responsible for life-threatening meningitis, especially in immunocompromised individuals. Although the gut–lung axis is known to regulate immune responses in respiratory infections, its role in cryptococcosis remains unclear. This study aimed to define the dynamic changes in the gut and lung microbiota and their relationship with host immunity during *C. neoformans* infection. Using a mouse model, we found that pulmonary infection induced significant dysbiosis in both the lung and gut microbiota, marked by decreased beneficial commensals and increased opportunistic pathogens. Integrated analysis showed these microbial shifts were closely associated with distinct immune responses: lung dysbiosis correlated with a strong IL-17-mediated pulmonary inflammatory response, while gut dysbiosis was linked to systemic immune activation in the spleen. Functional metagenomic prediction further revealed widespread disruption in microbial metabolic pathways, including energy metabolism and biosynthesis, in both sites. Importantly, a positive correlation was observed between lung and gut dysbiosis, indicating an interconnected gut–lung axis during cryptococcosis. These findings demonstrate that *C. neoformans* infection causes coordinated disruptions in microbiota and immunity across the gut–lung axis, underscoring the microbiome as a critical modulator of host response and suggesting potential avenues for microbiome-targeted therapies.

## 1. Introduction

*C. neoformans* is a pathogenic fungus, responsible for life-threatening meningoencephalitis, particularly in immunocompromised individuals [1,2,3]. Despite significant advances in antifungal therapy, cryptococcal meningitis continues to cause hundreds of thousands of deaths annually worldwide, particularly in the absence of timely diagnosis and intervention [4,5]. The persistently high mortality rate underscores the urgent need for a deeper understanding of its pathogenesis and the development of novel therapeutic strategies.

The traditional view of cryptococcosis had focused on the direct interaction between the pathogen and the immune system of the host. Early during Cryptococcus infection, alveolar macrophages and dendritic cells recognize the pathogen via pattern recognition receptors, triggering the production of pro-inflammatory cytokines such as TNF-α, IL-6, and IL-1β [6]. These cytokines promote neutrophil recruitment and activation, which play a dual role, limiting early fungal proliferation while potentially contributing to immunopathology [7]. As the infection progresses, the adaptive immune response is initiated, characterized by the differentiation of CD4^+^ T helper cells. A robust Th1 response, marked by IFN-γ production, is associated with protective immunity and effective fungal clearance, whereas a dominant Th2 response evidenced by elevated IL-4, IL-5, and IL-13 which is linked to disease exacerbation and poor clinical outcomes [8].

While these mechanisms have significantly advanced our understanding of host–Cryptococcus interactions, critical gaps remain. Notably, the roles of the gut microbiota and intestinal immunity in modulating systemic responses to cryptococcal infection are still poorly understood and represent an emerging frontier in fungal immunology [9,10,11]. The gut microbiome, in particular, exertes a profound influence on systemic immunity through the gut–lung axis, a bidirectional communication network [12,13]. This axis was exemplified by studies showing that the gut microbiota could shape pulmonary immune responses, such as the IL-17 response, during fungal challenges [14,15]. Crucially, a recent clinical study by Li et al. provided direct evidence that *C. neoformans* infection induced significant and lasting dysbiosis in the human gut microbiome, characterized by reduced beta diversity and enrichment of opportunistic bacteria [16]. This finding established that cryptococcosis was not merely a passive outcome of immunodeficiency but an active disruptor of the microbial ecosystem, which may in turn influence disease progression.

Antifungal treatments like fluconazole could cause gut dysbiosis, characterized by decreased microbial diversity, shifts in composition (e.g., increased Firmicutes and Proteobacteria, decreased Bacteroidetes and Ascomycota), and weakened colonization resistance, thereby increasing susceptibility to opportunistic and resistant infections [17,18]. Despite this clinical observation, critical knowledge gaps remained. The dynamics of the microbiome along the entire gut–lung axis during the course of *C. neoformans* infection were poorly characterized. To test this hypothesis, the present study employed a murine model of pulmonary cryptococcosis to comprehensively profile the temporal changes in both lung and gut microbiota following infection [18,19]. We integrated 16S rRNA sequencing, flow cytometric immune profiling, and metagenomic function prediction to delineate the interconnection between microbial shifts and host immunity. Our work aimed to provide a holistic view of the gut–lung axis dynamics in cryptococcosis, offering new insights into the role of microbiome as a disease modifier and revealing potential avenues for microbiome-targeted interventions.

## 2. Materials and Methods

### 2.1. Chemicals and Culture Media

Reagents were purchased from Thermo Fisher Scientific (Pittsburgh, PA, USA) unless otherwise specified. *C. neoformans* was cultured in YPD medium (containing 1% yeast extract, 2% bacteriological peptone, and 2% dextrose, with or without 2% agar), supplemented with chloramphenicol (Meilunbio, Dalian, Liaoning, China). Flow cytometry antibodies were procured from BD Biosciences and Thermo Fisher Scientific.

### 2.2. Animals and Ethics Statement

**Figure 1:**
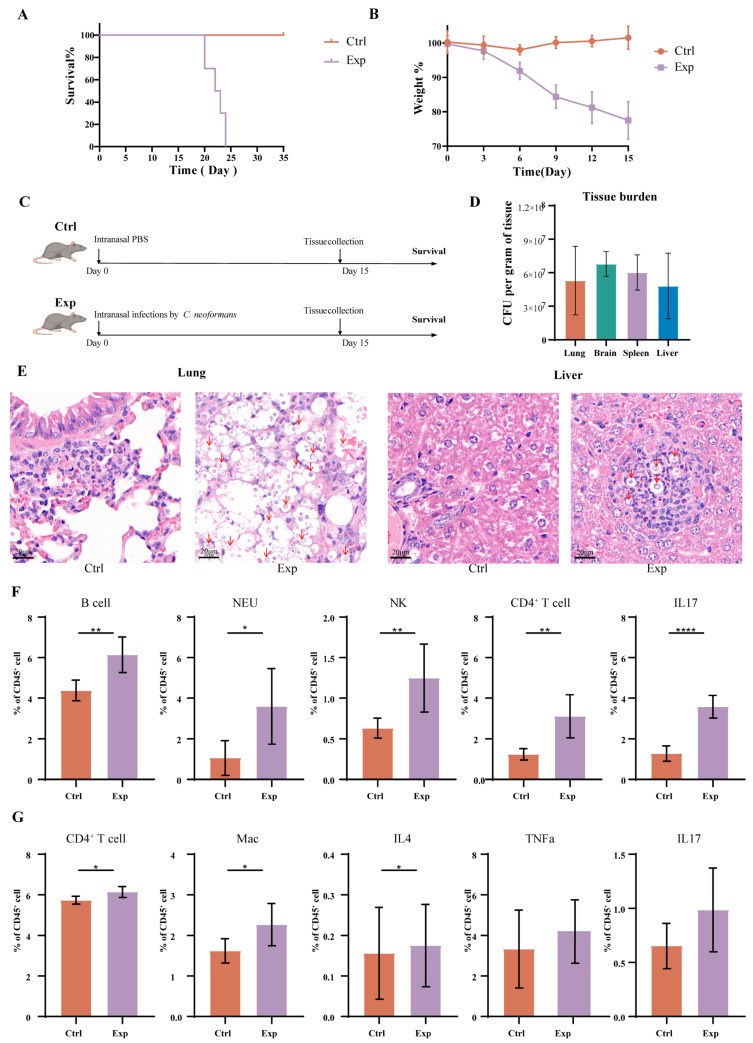
Establishment of a Murine Model of *C. neoformans* Infection. (**A**) Survival curve of mice following intranasal infection with *C. neoformans*. *p* < 0.001 (*p* values determined by Log-rank test) (**B**) Body weight changes in mice following intranasal infection with *C. neoformans* (data were presented as mean ± SEM). (**C**) Experimental grouping and design of the mouse study. (**D**) Fungal burden in organs of mice at 15 days post-infection with *C. neoformans*. (**E**) Histopathological examination of lung and liver tissues at day 15 post-infection. Lungs of *C. neoformans*-infected C57BL/6 mice exhibited severe granulomatous inflammation and tissue damage, whereas livers showed mild inflammatory responses. *C. neoformans* yeast cells (stained dark magenta with PAS) are indicated with red arrows. (**F**,**G**) Flow cytometry analysis of lung (**F**) and spleen (**G**) tissues (data were presented as mean ± SEM). The symbols *, **, and **** denote statistical significance compared to the control group at *p* < 0.05, *p* < 0.01, and *p* < 0.0001, respectively.

Female C57BL/6J mice, aged 7–8 weeks, were obtained from Jinan Laboratory Animal Technology (Jinan, China). Mice were confirmed by the vendor to be free of common murine pathogens, including pathogenic Helicobacter species. After a one-week acclimation period, mice were housed under standard conditions with free access to food and water. The experimental protocol was approved by the tab of Animal Experimental Ethical Inspection of Jinan Microecological Biomedicine Shandong Laboratory (approval code: 2025-55; approval date: January 2025). Animals were maintained in standard plastic cages with stainless steel lids at 25 ± 4 °C, 50 ± 5% relative humidity, under a 12 h light/dark cycle. Fourteen mice were randomly assigned to two groups: a control group (*n* = 7) and an experimental group (*n* = 7) (Figure 1A), monitored for approximately 30 days.

### 2.3. Fungal Culture

*C. neoformans* strain H99 was cultured on YPD agar plates for 2 days at 30 °C Colonies were used to inoculate liquid YPD medium. Yeast cells were harvested by centrifugation, washed twice with PBS, counted using a Neubauer chamber, and resuspended in PBS to the desired concentration.

### 2.4. Animal Infection Model

C57BL/6J mice (7–8 weeks old) were anesthetized and infected intranasally (i.n.) with a 1 × 10^5^ CFU inoculum in 50 μL PBS. Anesthesia was induced by intraperitoneal (i.p.) injection of 400 μL of 1.25% tribromoethanol.

### 2.5. Tissue and Feces Collection

At the endpoint, mice were euthanized by cervical dislocation under deep anesthesia. Mice were euthanized, and their surfaces were sterilized with 75% ethanol. The lungs, brains, livers, spleens, and kidneys were aseptically collected. Extreme care was taken during tissue collection to avoid contamination from the oropharynx or skin. Lung tissue and fecal samples intended for 16S rRNA sequencing were immediately snap-frozen in liquid nitrogen and stored at −80 °C.

### 2.6. Analysis of Fungal Burden in Tissues

Mice (*n* = 7 per group) were euthanized 15 days post-infection. The brain, liver, spleen, and lungs were homogenized in PBS. Serial dilutions of the homogenates were plated in duplicate on YPD agar containing 25 μg/mL chloramphenicol. CFUs were enumerated after 2–3 days of incubation at 30 °C.

### 2.7. Histology

Tissues were perfused with 4% paraformaldehyde, embedded in paraffin, and sectioned at 5 μm thickness. Sections were stained with periodic acid-Schiff (PAS) to visualize fungal cells and tissue morphology.

### 2.8. Flow Cytometry

Single-cell suspensions were prepared from murine lung and spleen tissues. Lung tissues were minced and digested enzymatically with a mixture of Liberase (5 μL/mL) and DNase I (10 μL/mL) in RPMI-1640 medium at 37 °C for 35 min with agitation. The resulting suspension was mechanically dissociated through a 70-μm cell strainer, washed with cold flow cytometry buffer, and centrifuged at 250× *g* for 5 min at 4 °C.

For immunophenotyping, approximately 2 × 10^6^ cells per sample were stained with a Fixable Viability Stain (BD Biosciences, San Jose, CA, USA, 564996) to exclude dead cells, followed by Fc receptor blockade. Cell surface staining was performed using a pre-titrated antibody cocktail against CD45 (BD Biosciences, 564279), CD4 (BD Biosciences, 740007), CD8a (BD Biosciences, 557959), CD19 (BD Biosciences, 557655), CD11b (BD Biosciences, 557396), Ly-6G (BD Biosciences, 740953), F4/80 (BD Biosciences, 565411), CD11c (BD Biosciences, 558079), NK-1.1 (BD Biosciences, 740853), and TCR-β (BD Biosciences, 746385).

For intracellular staining, cells were stimulated ex vivo with a leukocyte activation cocktail (BD Biosciences, 550583) for 4–6 h. After surface staining, cells were fixed and permeabilized using a commercial kit (BD Biosciences, 554714) and stained intracellularly with antibodies against IFN-γ (BD Biosciences, 554413), TNF-α (BD Biosciences, 566510), IL-17A (BD Biosciences, 564169), IL-4 (BD Biosciences, 554389), iNOS (Thermo Fisher Scientific, 414-5920-82), and ARG1 (Thermo Fisher Scientific, 25-3697-82). Data were acquired on a flow cytometer and analyzed using FlowJo software v11. The flow cytometry gating hierarchy is depicted in Supplementary Figure S1.

### 2.9. Statistical Analysis

Comparisons between two groups were performed using Student’s *t*-test. A *p*-value of less than 0.05 was considered statistically significant. All experiments were repeated at least twice.

## 3. Results

### 3.1. Host Morbidity and Systemic Dissemination Following C. neoformans Infection

Mice were randomly allocated into a control group (receiving intranasal PBS) and an experimental group (infected intranasally with *C. neoformans* in PBS) (Figure 1C). The infection model was lethal, with infected mice exhibiting a survival period of 20–25 days (Figure 1A). To characterize the host response during the established phase of infection, we monitored murine body weight and determined the fungal burden in major organs on day 15 post-infection, a timepoint selected to represent the mid-phase with robust and consistent pathological changes (Figure 1B,D).

PAS staining revealed systemic dissemination of *C. neoformans*, with extensive fungal colonization detected not only in the primary pulmonary site but also in distal organs such as the liver (Figure 1E). The lungs exhibited extensive granuloma formation, accompanied by marked inflammatory infiltration and notable tissue necrosis. The liver showed mild inflammatory changes with the presence of a few, small granulomas, indicating a less severe inflammatory response and fungal burden in peripheral organs.

Flow cytometric analysis of the pulmonary immune landscape revealed a response consistent with an active, IL-17-driven defense. We observed a significant influx of B cells, neutrophils, natural killer cells, and CD4^+^ T cells into the lungs (Figure 1F). Most notably, there was a marked elevation in the frequency of IL-17-producing cells, aligning with its known role in orchestrating early neutrophil recruitment and mucosal defense [20]. However, this robust IL-17 response occurred in the context of sustained high fungal burden and severe tissue inflammation (Figure 1E), a hallmark of immunopathology linked to detrimental outcomes. This suggests that in this model, the protective early IL-17 signaling may have transitioned into a dysregulated, pathogenic state.

Systemic immune activation was evident in the spleen, characterized by increased frequencies of CD4^+^ T cells, macrophages, and specifically M1-polarized macrophages. The cytokine profile mirrored a pro-inflammatory shift, with elevated levels of TNF-α and IL-17, alongside a moderate increase in the anti-inflammatory cytokine IL-4 (Figure 1G). This systemic elevation of IL-17 further supports its central role in the immune response to disseminated cryptococcosis and underscores the potential for a sustained, dysregulated signal to contribute to systemic immunopathology [21].

### 3.2. C. neoformans Infection Alters Microbial Community Composition in the Gut–Lung Axis

**Figure 2:**
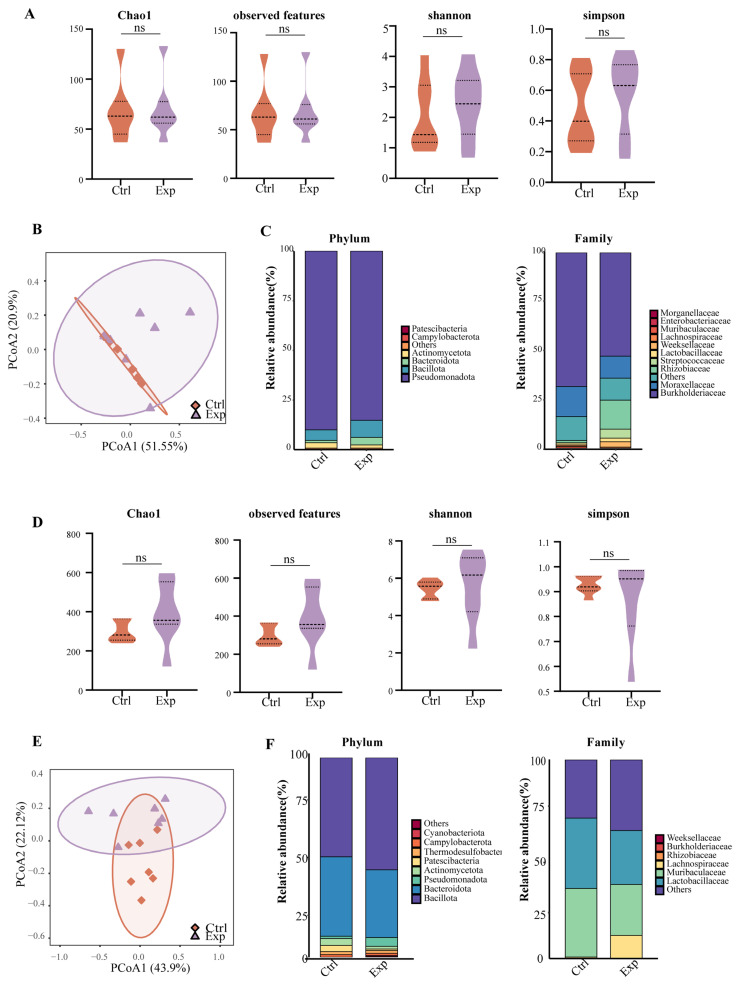
Microbiome analysis of lung and gut. (**A**,**D**) Alpha diversity of the (**A**) lung and (**D**) gut microbiome in mice. Boxplots represented Chao1 (richness), Observed Features (richness), Shannon (evenness and richness), and Simpson (evenness and richness) indices. Between-group differences were assessed using the Kruskal–Wallis test with Dunn’s post hoc correction. The dotted lines represented the quartiles and the dashed line represented the median. (**B**,**E**) Beta diversity of the (**B**) lung and (**E**) gut microbiome in mice. Principal Coordinate Analysis (PCoA) plot based on the Bray–Curtis dissimilarity matrix. Group separation was tested using PERMANOVA, as indicated in the legend. The circles represented the confidence ellipses (95% confidence intervals). (**C**,**F**) Bacterial relative abundance analysis of the (**C**) lung and (**F**) gut microbiome at different taxonomic levels. Bar plots showed mean relative abundance at the phylum and family levels. “ns” represented no significant difference.

Given the close association between microbiota, immunity, and disease progression, we profiled microbial communities along the gut–lung axis to assess the systemic impact of *C. neoformans* infection. Analysis of α-diversity, measured by species richness, showed no significant alteration in either lung or fecal samples during infection (Figure 2A,D), suggesting that the overall microbial load remained stable. However, a more nuanced picture emerged from β-diversity analysis, which assesses community structure. Notably, the composition of fecal microbial communities exhibited a more pronounced shift than that of lung communities post-infection (Figure 2B,E). This disparity indicated that cryptococcal infection induces niche-specific dysbiosis, with the gut microbiota demonstrating greater compositional sensitivity to systemic fungal challenge, potentially reflecting its role as a central immune modulator.

Further analysis of differentially abundant taxa revealed that infection perturbed key commensal populations in both sites. In the lungs, a primary site of infection, we observed a significant decrease in the abundance of Streptococcus (Bacillota) and Rhizobium (Proteobacteria) (Figure 2C). The reduction in pulmonary Streptococcus, some species of which can modulate host defense against respiratory pathogens [22], may represent a loss of potentially beneficial bacteria that could influence local immune homeostasis. Similarly, the decline in Rhizobium, an environmental genus detected in the lung microbiota, potentially indicating a loss of niche stability [23], might further contribute to an altered pulmonary microenvironment [24]. More strikingly, cryptococcal infection induced a significant reduction in the abundance of taxa belonging to the family Lachnospiraceae (Bacillota) at the distal gut site (Figure 2F). This observation is of particular relevance because Lachnospiraceae are major producers of immunomodulatory short-chain fatty acids (SCFAs), such as butyrate. These metabolites are crucial for maintaining intestinal epithelial barrier integrity, regulating anti-inflammatory responses, and educating systemic immunity [25]. Therefore, the depletion of these beneficial bacteria suggests that *C. neoformans* infection actively compromises a critical microbial source of immunoregulatory metabolites. This compromise may disrupt gut–lung axis communication and exacerbate systemic inflammation.

### 3.3. Analysis of Differentially Enriched Taxa in the Lung and Gut Microbiome

**Figure 3:**
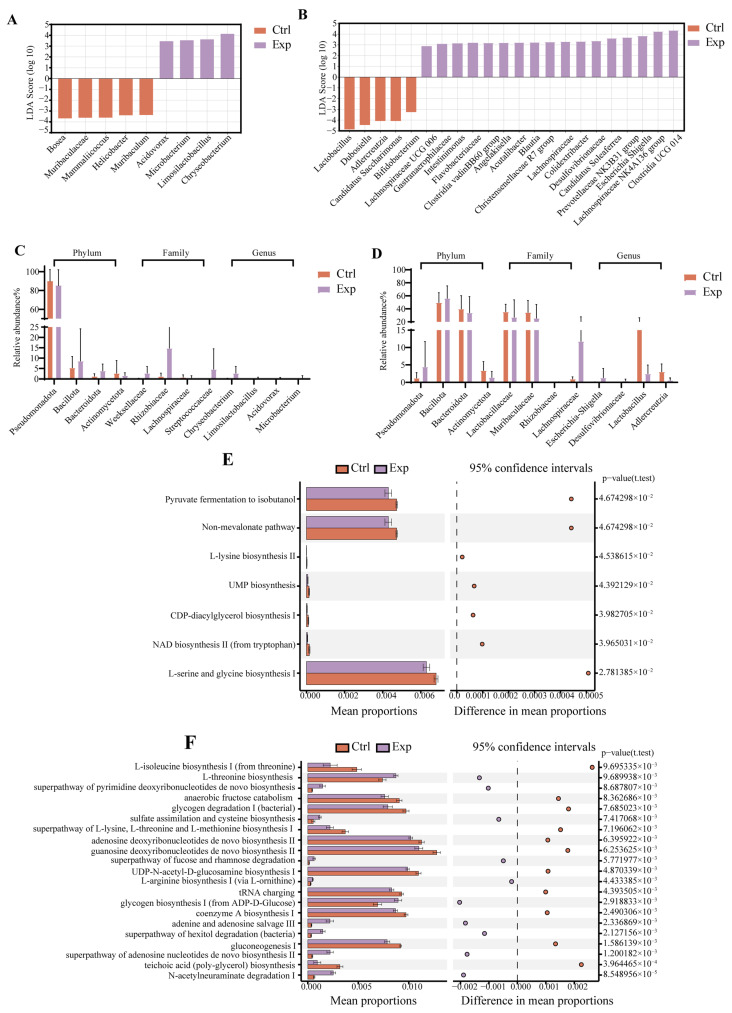
Differences in the lung and gut microbiome. (**A**,**B**) Linear discriminant analysis effect size (LEfSe) analysis at the genus level showing the differentially abundant microbiota between Ctrl and Exp in the (**A**) lung and (**B**) gut samples, respectively. Taxa enriched in the Ctrl group are shown in orange, and those enriched in the Exp group are shown in purple. The analysis was performed using LEfSe with an LDA score threshold of >2. (**C**,**D**) Relative bacterial abundance across taxonomic levels (phylum, family, genus) in lung and gut microbiome. (**E**,**F**) STAMP plot displayed KEGG pathways with significant differences in abundance (*p* < 0.05) as predicted by PICRUSt2 from 16S rRNA gene data for the lung (**E**) and gut (**F**) microbiome. All comparisons were based on a two-sided Welch’s *t*-test, with 95% confidence intervals shown.

LEfSe analysis revealed significant, niche-specific restructuring of bacterial communities in response to *C. neoformans* infection, with shifts involving both potentially detrimental and beneficial taxa. In the lungs, the microbial landscape was notably altered. We observed a significant enrichment of opportunistic pathogens, including Chryseobacterium, Microbacteriaceae, and Acidovorax (Figure 3A). The expansion of such taxa is consistent with a compromised pulmonary microenvironment during fungal disease. Concurrently, a notable increase was observed in the genus Limosilactobacillus (Figure 3C). As lactobacilli were known to modulate host immunity and have been implicated in gut–lung axis communication, their presence in the infected lung may represent a host-driven attempt to recruit or foster beneficial microbes to regulate local inflammation. In the gut, the dysbiosis was more complex, characterized by a simultaneous expansion of pathogenic potential and a counter-regulatory response. On one hand, we detected significant enrichment of known opportunistic pathogens, such as Flavobacteriaceae and Escherichia-Shigella [26], alongside Desulfovibrionaceae, a family of sulfate-reducing bacteria whose production of hydrogen sulfide can disrupt intestinal barrier function and promote inflammation [27]. This suggested an infection-induced impairment of gut homeostasis.

On the other hand, the gut microbiota also mounted a compensatory response. We observed significant enrichment of several families and genera renowned for producing short-chain fatty acids (SCFAs), including Lachnospiraceae, Intestinimonas, the Prevotellaceae NK3B31 group, Christensenellaceae, and the Christensenellaceae R-7 group [28,29,30] (Figure 3B). SCFAs like butyrate were potent anti-inflammatory metabolites that strengthen the gut barrier and systemically modulate immune responses, potentially serving to mitigate the systemic inflammation driven by cryptococcosis.

However, this beneficial shift was incomplete. A significant reduction occurred in other key health-associated taxa, including Lactobacillus, Dubosiella, Adlercreutzia, and Bifidobacterium [31] (Figure 3D). These genera contributed to host health through the production of lactate, SCFAs, and the phytoestrogen metabolite equol [32]. Their collective depletion indicated a substantial loss of core beneficial functions, which may undermine the stability of the gut ecosystem and its ability to regulate systemic immunity effectively.

### 3.4. Functional Profiling of the Lung and Fecal Microbiome

In the pulmonary microenvironment, PICRUSt2 analysis revealed a significant downregulation of multiple core metabolic pathways in infected mice compared to controls (Figure 3E). This included reductions in pathways critical for cellular energy and redox balance (NAD biosynthesis II and methylerythritol phosphate pathways), the metabolism of key one-carbon units (C1 compound oxidation), and the biosynthesis/degradation of immunomodulatory amino acids (L-tryptophan degradation and L-serine/glycine biosynthesis). The coordinated suppression of these diverse metabolic modules strongly suggested a broad functional impairment of the resident lung microbiota [33,34]. This functional suppression reflected the active displacement or impairment of commensal bacteria, suggesting a consequent loss of microbial metabolites crucial for pulmonary epithelial and immune homeostasis [35,36].

In contrast, the gut microbiota exhibited a distinct and aggressive metabolic reprogramming in response to systemic infection (Figure 3F). We observed a significant weakening of fundamental anabolic and energy-generating pathways central to bacterial autonomy, including homolactic fermentation, coenzyme A biosynthesis, gluconeogenesis, glycolysis, the TCA cycle, and nucleotide biosynthesis. This downregulation of core cellular maintenance metabolism coincided with a marked upregulation of pathways dedicated to the degradation of host-derived substrates, such as fucose, rhamnose, and purines [37,38]. Following infection, the gut microbiota underwent a hallmark dysbiotic shift. PICRUSt2-based inference suggested that this shift might involve a metabolic reconfiguration toward scavenging host-derived nutrients [39]. This predicted shift toward a parasitic nutritional mode represents a plausible mechanistic contributor to the observed pathology, as it could facilitate barrier damage and systemic inflammatory spillover. However, the present data cannot determine whether such a mode is a driving cause or merely a parallel consequence of the systemic inflammatory state. The PICRUSt2 predictions indicated a potential systemic shift toward a parasitic nutritional mode following infection, with potential consequences of exacerbated barrier damage, inflammation, and systemic release of pro-inflammatory molecules; however, this hypothesis necessitates confirmation via integrated multi-omics validation. Therefore, future work integrating metabolomic data is crucial to validate this prediction and to investigate specific axis communication mechanisms, such as the translocation of microbial metabolites.

Collectively, the contrasting functional alterations, specifically metabolic suppression in the lung and metabolic parasitism in the gut, provided a mechanistic basis for the observed taxonomic dysbiosis. These alterations demonstrated that the infection fundamentally redirected microbial community function. The functional decay in the lung potentially weakened local defense, while the host-targeting metabolism in the gut likely exacerbated systemic inflammation. These findings illustrated a functional interplay within the gut–lung axis that promoted disease progression.

### 3.5. Correlation Between Key Bacterial Taxa and Host Immune Indices

**Figure 4:**
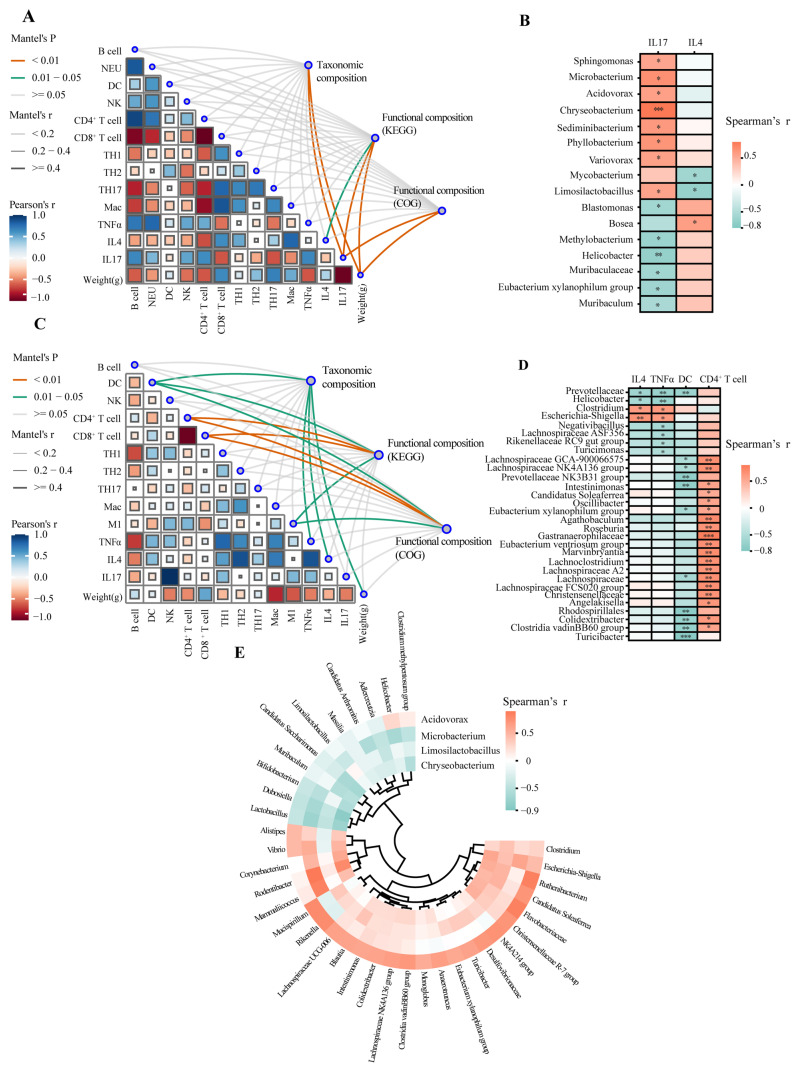
Interplay between the murine lung, gut microbiome and host immune characteristics. (**A**,**C**) Mantel test of the lung immune profile and the taxonomic composition in the lung (**A**) and gut (**C**). Shades of red indicated positive covariation, while shades of blue indicated negative covariation. The strength of correlation between the two distance matrices was classified based on the Mantel’s r value: strong (|r| > 0.4), moderate (0.2 ≤ |r| ≤ 0.4), weak (|r| < 0.2). The strength of correlation between the two variables was classified based on the Pearson’s r value: strong (|r| > 0.4), moderate (0.2 ≤ |r| ≤ 0.4), weak (|r| < 0.2). (**B**,**D**) Heatmap of spearman correlations between host immune parameters of lung and differentially represented bacterial taxa in the lung (**B**) and gut (**D**). Positive correlations (orange) indicated that higher relative abundance of a taxon is associated with higher levels of an immune parameter, while negative correlations (blue) indicate an inverse relationship. The strength of correlation was interpreted based on the absolute value of |r| as follows: strong (|r| > 0.7), moderate (0.3 < |r| < 0.7), and weak/no association (|r| < 0.3). (**E**) Cross-organ association of differentially abundant bacterial taxa assessed by Spearman correlation analysis between murine lung and gut. The symbols *, **, and *** denote statistical significance compared to the control group at *p* < 0.05, *p* < 0.01, and *p* < 0.001, respectively. The items without asterisks indicated no significant difference.

Correlation analysis integrating microbial and immunological datasets revealed structured, axis-specific interactions, delineating how local and systemic inflammation are linked to niche-specific dysbiosis during *C. neoformans* infection. In the lungs, the composition of the microbiota showed a strong and specific association with the levels of IL-17 (Figure 4A,C). This observation aligned with the canonical role of IL-17 in mucosal immunity. The data indicated that the IL-17-driven response acted as an environmental filter shaping the post-infection lung microbiota, potentially establishing potential interconnections between inflammation and bacterial community structure. Conversely, gut dysbiosis correlated with systemic immune parameters from the spleen, not local pulmonary signals, supporting its origin from systemic inflammatory spillover rather than direct fungal effects. Significant correlations were identified with the frequencies of antigen-presenting dendritic cells, adaptive immune populations (CD4^+^ and CD8^+^ T cells), and the levels of both TNF-α and IL-4 cytokines (Figure 4B,D). This pattern indicates that systemic immune activation, reflected in the splenic compartment, is a major driver of remote gut microbial restructuring [40].

Furthermore, we identified a concrete microbiological signature of this gut–lung axis communication. A parallel increase in opportunistic pathogens from the Escherichia-Shigella group was observed in both the lung and gut compartments (Figure 4E). The synchronous expansion of this pathogenic taxon provided direct evidence for a sequential gut–lung axis effect, supporting a model where systemic inflammation-driven gut dysbiosis facilitates pathobiont expansion that may exacerbate inflammation in distant sites, including the lungs. The detection of Escherichia-Shigella as a shared taxon in the lung and intestinal niches aligned with the concept of bacterial translocation via the gut–lung axis. This mechanism was exemplified by Staphylococcus aureus, which literature indicated that Staphylococcus aureus could survive intracellularly within gut mucosal phagocytes, including monocytes and macrophages. These immune cells transport the bacteria systemically, releasing them into lung tissue and thereby facilitating pulmonary colonization [41].

In summary, correlation analysis showed organ-specific immune–microbe coupling, with lung dysbiosis linked to local IL-17 and gut dysbiosis to systemic activation. The conserved expansion of Escherichia-Shigella offered tangible evidence of axis crosstalk, positioning the disrupted gut microbiome as a potential amplifier of cryptococcal disease pathophysiology.

## 4. Discussion

Emerging research has underscored the gut microbiome as a critical modulator of systemic immunity and disease outcomes. Aligning with this paradigm, a foundational clinical study demonstrated that cryptococcal meningitis itself induced significant gut microbial dysbiosis in patients, characterized by altered community structure and specific taxonomic shifts, including an enrichment of Firmicutes and a depletion of Prevotella [16]. This pivotal observation positioned the gut microbiome as an active participant in cryptococcal disease, which prompted our investigation into the underlying host–microbe interactions within the gut–lung axis using a murine model.

Our experimental findings corroborated and extended this clinical insight. It was important to note that this model investigated primary, progressive pulmonary cryptococcosis. While *C. neoformans* is an opportunistic pathogen, the use of immunocompetent mice allows for the dissection of host–pathogen–microbiota interactions in the absence of pre-existing major immunodeficiency, providing a clear view of the infection’s direct impact. We confirmed that pulmonary infection with *C. neoformans* induced severe local and systemic inflammation, accompanied by significant, niche-specific dysbiosis in both the lung and gut microbiomes. Crucially, these shifts transcended taxonomy. Functional prediction analyses revealed a profound metabolic reprogramming. The lung microbiota exhibited a broad suppression of core metabolic pathways, while the gut microbiota shifted toward a parasitic nutritional mode, scavenging host-derived nutrients at the expense of its own metabolic independence [42]. This indicated that the infection actively compromised the homeostatic and metabolic functions of commensal microbial communities. Furthermore, the contribution of infection-associated anorexia and weight loss to the observed gut microbial dysbiosis cannot be ruled out and represents an important area for future investigation. Despite diligent efforts to standardize and optimize our aseptic tissue collection protocol, an inherent risk of airborne or contact contamination cannot be entirely eliminated. Future studies incorporating histological stains or bacterial culture are needed to confirm the viability and spatial localization of these bacterial communities within infected tissues.

Integrated correlation analysis clearly delineated the interconnection between these dysbiotic events and host immunity. We established an organ-specific coupling, where pulmonary dysbiosis was directly linked to the local, IL-17-driven inflammatory response, and gut dysbiosis correlated strongly with broader systemic immune activation markers. It was important to note that the association between the IL-17 response and microbial dysbiosis identified here was correlative. Definitive proof of a causal role for IL-17 signaling in shaping the microbiota during cryptococcosis required future interventional studies using models such as IL-17 or IL-17R deficient mice. This compartmentalized association supported a mechanistic model in which local fungal infection and systemic inflammatory spillover drove microbial disturbances in the lung and gut, respectively. The discovery of a synchronous expansion of opportunistic pathogens, such as Escherichia-Shigella, in both sites provided tangible evidence for a functional gut–lung axis, suggesting a potential vicious cycle. In this cycle, systemic inflammation likely disrupted gut barrier integrity and microbiota, which may then have exacerbated pulmonary inflammation through mechanisms like bacterial translocation or the dissemination of microbial-derived inflammatory signals, ultimately worsening disease outcomes. While our correlative data support this interpretation, further mechanistic studies are required to confirm this sequence of events and its contribution to disease outcomes.

Within this disrupted ecosystem, the gut microbiota displayed a paradoxical response, highlighting its complex dual role during infection. We observed a concurrent enrichment of both beneficial short-chain fatty acid (SCFA)-producing families and harmful pathobionts. The former may have represented a compensatory, host-protective feedback mechanism aimed at restraining inflammation. However, the overarching landscape was marked by the significant loss of other key commensals and the dominance of pathobionts, which likely tipped the functional balance toward a net detrimental state. This imbalance may have critically impaired the gut’s capacity to produce immunoregulatory metabolites while amplifying pro-inflammatory signals, thereby fueling the systemic inflammation characteristic of severe cryptococcosis.

In conclusion, our study delineated potential interconnections within the gut–lung axis during *C. neoformans* infection. The infection and ensuing inflammation induced functionally detrimental dysbiosis in both the lung and gut. These microbial disturbances, in turn, were positioned to amplify local and systemic immune pathology. This framework not only deepens our understanding of cryptococcosis pathogenesis but also positions the gut microbiome and its metabolic output as potential novel therapeutic targets. Future interventions aimed at preserving or restoring microbial homeostasis and function could represent a promising adjunctive strategy to dampen detrimental inflammation and improve outcomes in this lethal fungal disease.

## 5. Conclusions

In summary, our findings established that systemic infection with *C. neoformans* induced profound and functionally consequential dysbiosis in both the lung and gut microbiota. This dysbiosis was characterized by a significant expansion of opportunistic pathogens in each niche, a pattern consistent with prior observations in clinical cryptococcosis. Crucially, the positive correlation between pulmonary and intestinal ecological disruption provided direct experimental evidence for a functionally interconnected gut–lung axis during fungal disease. Furthermore, the close and organ-specific association of these microecological shifted with distinct host immune responses, specifically local IL-17-driven inflammation in the lung and systemic immune activation as reflected by the gut, definitively highlighted the microbiota as a pivotal disease modifier in cryptococcal pathogenesis. Collectively, this work delineated a pathogenic circuit in which infection-driven inflammation disrupts microbial communities, and the resultant dysbiosis, particularly in the gut, may in turn amplify systemic inflammation, thereby exacerbating disease outcomes.

## Data Availability

The sequencing data generated in this study have been deposited in the China National Center for Bioinformation (CNCB) under accession number PRJCA050145 (accessed on 20 February 2026). These data are publicly accessible via the BIG Sub system at https://ngdc.cncb.ac.cn/gsub/submit/bioproject/subPRO073586/overview.

## Supplementary Materials

The following supporting information can be downloaded at: https://www.mdpi.com/article/10.3390/jof12030163/s1, Figure S1: Flow Cytometry Gating Strategy. Concerning the following immune cell populations: lymphocytes, single cells, leukocytes, B cells, T cells (including CD4^+^ and CD8^+^ T cells, as well as Th1, Th2, and Th17 lineages), dendritic cells, neutrophils, macrophages (both M1 and M2 phenotypes), and the cytokines IL-4, TNF-α, IFN-γ, and IL-17.
