# Supplementary material for: Dysbiosis of the Gut–Lung Axis and Its Immune Correlates During Pulmonary Cryptococcus neoformans Infection
Source: J Fungi (Basel). 2026 Feb 25;12(3):163. doi: 10.3390/jof12030163 (PMC13027605; doi:10.3390/jof12030163)
Supplement: Supplementary file 1 [file jof-12-00163-s001.zip › jof-4086627-Supplementary.pdf]

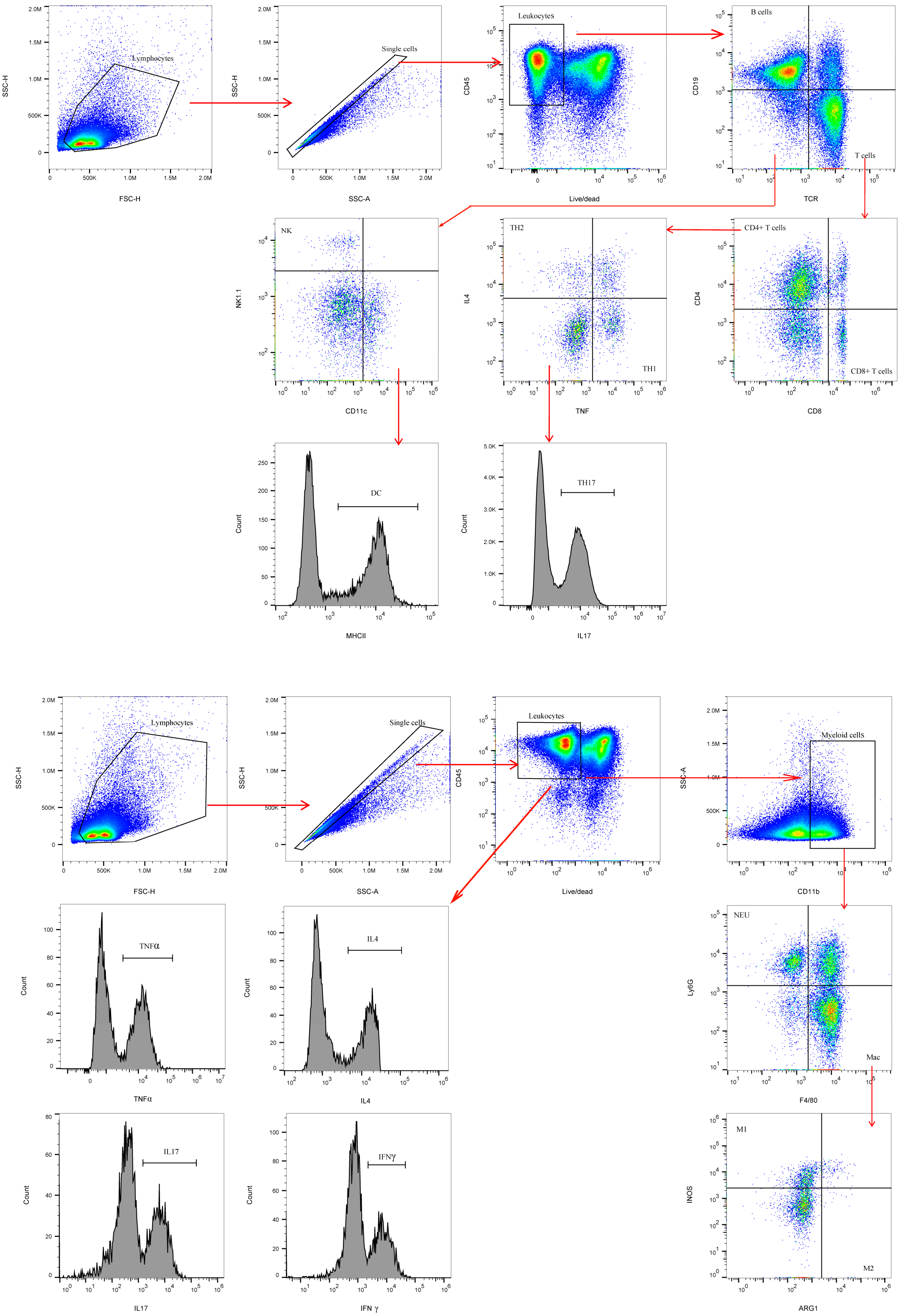

https://doi.org/10.3390/jof12030163

Supplementary Figure S1. Flow Cytometry Gating Strategy. Concerning the following immune cell populations: lymphocytes, single cells, leukocytes, B cells, T cells (including $CD4^+$ and $CD8^+$ T cells, as well as Th1, Th2, and Th17 lineages), dendritic cells, neutrophils, macrophages (both M1 and M2 phenotypes), and the cytokines IL-4, TNF-α, IFN-γ, and IL-17.
